# A Novel RAA Combined Test Strip Method Based on Dual Gene Targets for Pathogenic *Vibrio vulnificus* in Aquatic Products

**DOI:** 10.3390/foods12193605

**Published:** 2023-09-28

**Authors:** Wenyue Liu, Guangying Zhang, Di Xu, Jingqin Ye, Ying Lu

**Affiliations:** 1 College of Food Science and Technology, Shanghai Ocean University, Shanghai 201306, China; yul777lwy@163.com (W.L.); zzz_guangying@163.com (G.Z.); xd199702@163.com (D.X.); 2 Laboratory of Quality and Safety Risk Assessment for Aquatic Products on Storage and Preservation (Shanghai), Ministry of Agriculture, Shanghai 201306, China; 3 State Key Laboratory of Pathogen and Biosecurity, Beijing Institute of Biotechnology, Beijing 100071, China; yejq0922@163.com; 4 Marine Biomedical Science and Technology Innovation Platform of Lingang Special Area, Shanghai 201306, China

**Keywords:** *V. vulnificus*, recombinase-aided amplification, test strip, *vvhA*, *gyrB*

## Abstract

*Vibrio vulnificus* can cause disease in aquatic animals and humans, therefore, rapid and simple field detection of pathogenic *V. vulnificus* is important for early disease prevention. In this study, a novel recombinase-aided amplification (RAA) combined test strip with double T-lines (RAA-TS-DTL) was developed for the rapid detection of *V. vulnificus* in aquatic products. Pathogenic *V. vulnificus* was detected using the virulence *vvhA* gene and the housekeeping gene *gyrB* gene as the dual target of the test strip. The RAA-TS-DTL method showed 100% specificity for *V. vulnificus*, and no cross-reaction was observed with *Vibrio* spp. or other bacteria (*n* = 14). Furthermore, sensitive detection of *V. vulnificus* in oysters was achieved. The LODs of the *gyrB* and *vvhA* genes were 6 CFU/mL and 23 CFU/mL, respectively, which was about five times higher than that of the commercial test strip. The method was validated with spiked samples (*n* = 60) of fish, shrimp and oyster. The consistency between RAA-TS-DTL and the traditional culture method was 97.9%. In addition, the entire process of detection, including preparation of the sample, could be completed within 50 min. Our results indicated that the developed RAA-TS-DTL was a reliable and useful tool for rapid screening or on-site detection of pathogenic *V. vulnificus* in aquatic products and aquaculture water.

## 1. Introduction

*Vibrio vulnificus* is a Gram-negative bacterium that is widely distributed in ocean, river and aquatic products, and can accumulate in shellfish such as oysters [1]. It causes acute gastroenteritis, mainly through the consumption of undercooked seafood products [2]. It was reported that from 2002 to 2007, 92.8% of 180 people with *V. vulnificus* infection had eaten raw oysters prior to the onset of symptoms [3]. In addition, life-threatening infections can also occur when damaged skin is exposed to seawater or seafood contaminated with *V. vulnificus* [4]. When a person with impaired or low immunity is infected with *V. vulnificus*, it is easy to induce septic shock and multiple organ failure, resulting in a case fatality rate of up to 50% [3]. With global warming and the rise in sea temperature, *V. vulnificus* has proliferated and appeared widely in seafood products, leading to a gradual increase in the number of people infected with *V. vulnificus* [5].

Currently, *V. vulnificus* is detected by traditional morphological, immunological and molecular biological methods. The traditional morphological method, as a conventional detection method of *V. vulnificus*, is easy to operate. However, it is time-consuming, and it can be difficult to distinguish the *Vibrio* species with a similar morphology. The most commonly used immunological methods are the enzyme-linked immunosorbent assay (ELISA) and immunochromatographic test strips (ICTSs). They are all based on the specific reaction between the antigen and the antibody to realize detection of *V. vulnificus*. Wangman et al. [6] established a sensitive ELISA method achieving a detection of 1 CFU/mL *V. vulnificus* in oysters, but it required a long time (>3 h) and was not suitable for rapid field testing. Because of its simple and rapid operation, ICTS technology has been widely used in the field of rapid detection of *Vibrio* and other pathogens. For example, Jadeja et al. [7] established an ICTS method using a species-specific anti-H monoclonal antibody, which was able to detect 10 CFU/mL of *V. vulnificus* in oysters. Although this detection method is fast (5 min), it was necessary to preculture it for about 6 h to enrich *V. vulnificus* before detection.

The molecular biology methods used for *V. vulnificus* were originally polymerase chain reaction (PCR) and quantitative PCR (qPCR) realized by the amplification of target genes such as virulence genes or conserved genes. D’Souza et al. [8] established a SYBR Green qPCR assay based on the housekeeping gene *gyrB* of *V. vulnificus*, which could detect 10 CFU/mL of *V. vulnificus* in a pure culture broth. Meanwhile, Li et al. [9] developed a quantitative real-time PCR (qRT-PCR) method based on the *vvhA* gene to detect *V. vulnificus* at 15 CFU/mL. These PCR methods are highly sensitive and accurate, but require specialized equipment and trained operators. Because rapid DNA amplification can be achieved with the only requirement being a constant-temperature heating device, isothermal amplification techniques of *V. vulnificus* such as loop-mediated isothermal amplification (LAMP), recombinase polymerase amplification (RPA) and recombinase-aided amplification (RAA) have been developed successfully. Tian et al. [10] developed a LAMP method that could detect 10 fg/μL of *V. vulnificus* in a culture solution by amplification at 65 °C for 30 min. Yang et al. [11] realized the detection of 2 CFU/mL of *V. vulnificus* in spiked shrimp samples through the RPA principle at 37 °C for 35 min. Compared with LAMP, the reaction temperatures of RPA or RAA are closer to room temperature, therefore they are more suitable for developing rapid detection methods in the field. Fang et al. [12] developed a RAA combined test strip method based on the virulence *ctxA* gene, which could detect 46 CFU/mL of *Vibrio cholerae*. Meanwhile, Xiao et al. [13] established a combined RAA–CRISPR method, achieving the detection of 2 copies of *V. vulnificus* on the basis of the *vvhA* gene within 40 min. However, the RAA–CRISPR method requires a fluorescence excitation device, whereas a test strip is more suitable for on-site tests, as it allows direct color development such as colloidal gold, magnetic beads or colored microspheres. Early identification and detection of *V. vulnificus* in aquatic products can reduce the infection rate of *V. vulnificus* in humans. Therefore, it is necessary to develop rapid and accurate detection methods for *V. vulnificus*.

In order to develop a simple and accurate on-site detection method for pathogenic *V. vulnificus*, in this study, a detection system based on RAA combined with ICTS was constructed using the virulence gene *vvhA* and the housekeeping gene *gyrB* as targets. Primers were designed for each gene and modified with a fluorescent substance, biotin and digoxin, respectively. Their RAA products were used as samples for ICTS detection of *V. vulnificus*. Test strips with double test lines (TS-DTL) were constructed to achieve on-site detection, where the T_1_ line was coated with streptavidin (SA) to detect the *gyrB* gene, while the T_2_ line was coated with FITC monoclonal antibody (mAb) to capture pathogenic *vvhA* gene. In addition, multi-branched gold nanoparticles (MBGNP) coupled with digoxin mAb were used as probes to capture the dual genes. The specific and sensitive detection of pathogenic *V. vulnificus* in foods was realized within 50 min, including pretreatment of the sample. This study provides a new ideal and tool for rapid screening and on-site detection of *V. vulnificus* in aquatic products.

## 2. Materials and Methods

### 2.1. Bacteria Culture and Extraction of Genomic DNA 

Eighteen bacteria were used in this study, including four strains of *V. vulnificus* (ATCC 27562, X3, S4 and M16), six strains of other *Vibrio* species and eight strains of non-*Vibrio* bacteria, as listed in Table 1. All bacteria were incubated in Luria–Bertani broth (LB, Land Bridge Technology Co., Ltd., Beijing, China) at 37 °C on a shaker for 8 h. *V. vulnificus* ATCC 27562 was diluted from 10^8^ to 1 CFU/mL with phosphate-buffered saline (PBS), and the bacterial solution was quantified using the plate count method. The genomic DNA of *V. vulnificus* ATCC 27562 was extracted according to the guide of the DNA extraction kit (Tiangen Biotech Co., Ltd., Beijing, China). The DNA of other bacteria was extracted by boiling for 10 min. The concentration obtained for each DNA was calculated by the absorbance at 260 nm. The DNA’s purity was analyzed in 2% agarose gel by agarose gel electrophoresis (AGE). All DNA was stored at −20 °C until use.

### 2.2. Design and Synthesis of the Primers for RAA

The *gyrB* and *vvhA* genes were selected as the detection targets of *V. vulnificus.* Five published *gyrB* gene sequences (GenBank Nos. EU118215.1, CP012881.1, EU118216.1, MN540397.1 and EU118204.1) and *vvhA* gene sequence (GenBank No. KF255334.1) were individually downloaded from NCBI. MEGA-X was used to compare the gene sequences of *gyrB* to obtain conservative sequences, and the comparison result was shown in Figure S1. Ten pairs of primers for the *vvhA* gene (Table 2) and the *gyrB* gene (Table 3) were separately designed by Primer Premier 5.0 (Premier Biosoft, Palo Alto, CA, USA). According to the preliminary reaction system recommended in the manufacturer’s instructions, basic commercial RAA kits (China Jiangsu Qitian Biotechnology Co., Ltd., Wuxi, China) were used to screen the primers that were suitable for the test strips. Namely, after 25 μL of the buffer, 2 μL of the forward and reverse primers, and 17.5 μL of purified water were mixed, 2.5 μL of a Mg(CH_3_COO)_2_ solution and 1 μL of DNA were added, and the reaction was kept at 37 °C for 30 min. The obtained RAA products were analyzed by electrophoresis in 2% agarose gel. On the basis of the screening results, Group 2 primers of the *vvhA* gene and Group 10 primers of the *gyrB* gene with good amplification efficiency were selected for synthesis and labeling, as shown in Table 2 and Table 3. The specificity analysis data of two primer pairs were shown in Figures S2 and S3. The primers of Group 10 were labeled with biotin and digoxin, and the primers of Group 2 were labeled with FAM and digoxin from Sangon Biotech Ltd. (Shanghai, China).

### 2.3. Preparation of the MBGNP Probe

Multi-branched gold nanoparticles (MBGNP) were prepared by the method described by Lu et al. [14] with slight modifications. Briefly, 50 μL of a 10% chloroauric acid solution and 50 mL of ultrapure water were heated to boiling in a water bath with vigorous stirring. Subsequently, 2 mL of a 1% trisodium citrate solution was added rapidly into the boiling solution. When the solution’s color gradually changed from colorless to burgundy and remained stable, the solution was cooled down to room temperature and used as seeds. Next, 50 μL of a 10% chloroauric acid solution, 0.89 mL of gold seeds and 1.32 mL of a 1% trisodium citrate solution were added to 50 mL of ultrapure water with stirring under heating to 50 °C. Then 12 mL of 30 mM hydroquinone was added to the solution and stirred continuously for 30 min at 25 °C until the color of the solution turned dark blue. The obtained MBGNP solution was stored at 4 °C until further use.

In line with the method described by Lu et al. [14], the size and morphology of the MBGNP were measured with a high-resolution transmission electron microscope (TEM; H-7800, Tokyo, Japan). The absorption peaks of the MBGNP were observed by a UV-visible spectrophotometer, and the hydrodynamic size distribution of the MBGNP was analyzed by scientific dynamic light scattering (DLS).

The MBGNP probe was prepared by modifying digoxin mAb on the surface of the MBGNP. Briefly, 1 mL of MBGNP was mixed with 4 μL of a 0.2 M K_2_CO_3_ solution, and 16 μg of digoxin mAb was subsequently added and reacted at 25 °C for 2 h. Next, 50 μL of a 10% (*w*/*v*) bovine serum albumin (BSA) solution was added and mixed for 30 min at 25 °C. The resulting mixture was washed twice with 20 mM PBS containing 1% (*w*/*v*) BSA and 0.5% (*v*/*v*) Tween-20 (PBS-BT). After centrifugation at 3000 rpm for 10 min, the precipitate was resuspended in 500 μL of a PBS-BT buffer containing 5% (*w*/*v*) sucrose and 0.05% (*w*/*v*) sodium azide, which was used as the MBGNP probe and stored in a refrigerator at 4 °C.

### 2.4. TS-DTL Preparation and Detection Procedure

For the preparation of dual detection line test strips, 2 mg/mL of FITC mAb and 0.5 mg/mL of SA in PBS sprayed onto a nitrocellulose (NC) membrane were used as the T_1_ and T_2_ lines, and 0.3 mg/mL GAM IgG was coated as the control line (C line) of each test strip (TS). After the NC membranes were dried at 37 °C for 2 h, the TS was constructed by fixing the sample pad, the binding pad, the NC membrane and the absorbent pad to the bottom plate. Then the TS with double-T lines (TS-DTL) was obtained by cutting the NC membrane into strips 5 mm in width, which were stored in aluminum foil bags at room temperature. For the detection of *V. vulnificus*, 20 μL of RAA amplification products (10 μL *vvhA* and 10 μL *gyrB*) were mixed with 80 μL of a chromatographic buffer, which was prepared by mixing 12 μL of the MBGNP probe and 8 μL of PBS-BT. Subsequently, 100 μL of the mixture was added onto the sample pad of the TS-DTL and allowed to flow for about 10–15 min.

### 2.5. Optimization of the Reaction Conditions of RAA-TS-DTL

DNA extracted from *V. vulnificus* ATCC 27562 was used as the amplification template to optimize the reaction temperature (15, 25, 30, 35, 37, 39, 41 and 55 °C) and time (5, 10, 15, 20, 25 and 30 min) of RAA. Furthermore, 10 μg of digoxin mAb with different MBGNP pH values (6.5, 7.0, 7.5, 8.0 and 8.5) were used to prepare the MBGNP probes, and the absorbance values at 460 nm were determined by a microplate reader [15]. In addition, different concentrations of FITC mAb (1.0, 1.5, 2.0 and 2.5 mg/mL) in 10 mM PBS, and various amounts of digoxin mAb (8, 16 and 32 μg) were chosen to optimize the detection conditions of TS-DTL. PBS (pH 7.4), Tris-HCl (pH 6.8), a carbonate-buffered saline solution (CBS, pH 9.6), a borate-buffered saline solution (BBS, pH 9.0) and pure water were individually used to optimize the chromatographic buffer of the TS-DTL.

### 2.6. Specificity of RAA-TS-DTL for V. vulnificus

The RAA amplification products of *vvhA* and *gyrB* genes from the 18 strains of bacteria listed in Table 1 were used as test samples. The mixture of 20 μL of the amplification product samples and 80 μL of the chromatographic buffer was tested according to the procedure described in Section 2.4.

### 2.7. Sensitivity of RAA-TS-DTL for V. vulnificus in Oyster

Fresh oyster samples spiked with *V. vulnificus* were prepared according to the methods described by Fang et al. [12] and Sun et al. [16]. Briefly, 10 g of oyster was soaked in 75% ethanol for 10 min. After washing three times with ultrapure water, the oyster muscle was homogenized in 10 mL PBS. The obtained homogenate was incubated in LB agar at 37 °C for 1 day to check the sterility of the oyster samples. Subsequently, 100 μL of different concentrations of *V. vulnificus* from 1 to 10^6^ CFU/mL were added individually to the sterile oyster homogenate to prepare spiked real food samples. After incubation at 37 °C for 2 h, the spiked samples were centrifuged at 200× *g* for 5 min, and the obtained supernatants were centrifuged again at 18,000× *g* for 5 min. Next, the precipitates were collected and resuspended in 100 μL of PBS. Genomic DNA extracted from the spiked samples was used as templates for the RAA and PCR reactions, respectively. The PCR primers listed in Table 2 and Table 3 were used for the PCR, and the obtained PCR products were analyzed by 2% AGE.

In addition, the RAA products were detected separately by the developed TS-DLS and a commercial test strip. The qualitative sensitivity of RAA-TS-DTL was determined by the sample’s concentration when the colored T lines disappeared. Quantitative detection was achieved with an image of RAA-TS-DTL taken by a smartphone. The grayscale values of the T_1_ and T_2_ lines were quantified by Image J software. Combined with the fitted regression formula of the quantitative curve, the quantitative limit of detection (LOD) of RAA-TS-DTL could be calculated according to the formula G_m_ = G_0_ + 3 × SD, where G_m_ (namely LOD) is the minimum detectable grayscale value, G_0_ is the grayscale value of the negative sample, and SD is the standard deviation of negative sample.

### 2.8. Accuracy Evaluation of RAA-TS-DTL

According to the method of Pedrosa et al. [17], the *V. vulnificus* solution was processed with PMAxx. Briefly, the bacterial solution was mixed with 40 μM PMAxx, incubated in the dark for 5 min and exposed for 15 min on an ice box 20 cm away from a 500 W halogen lamp. The processed *V. vulnificus* solutions of 10^2^, 10^3^ and 10^6^ CFU/mL were randomly added to 10 g of each aquatic product (refer to Section 2.7 for the preparation of contaminated samples). Three types of aquatic products including crucian carp (*Tilapia*, *n* = 20), white prawns (*Litopenaeus vannamei*, *n* = 30) and oysters (*Crassostrea gigas*, *n* = 10) were used as spiked food samples. These samples were detected by RAA-TS-DTL and PCR-AGE, respectively. Ultrapure water was used as a negative sample in the PCR-AGE method. On the other hand, each sample was tested according to a national standard culture method of China (GB 4789.44-2020).

## 3. Results

### 3.1. Principle of RAA-TS-DTL for the Detection of V. vulnificus

**Figure 1:**
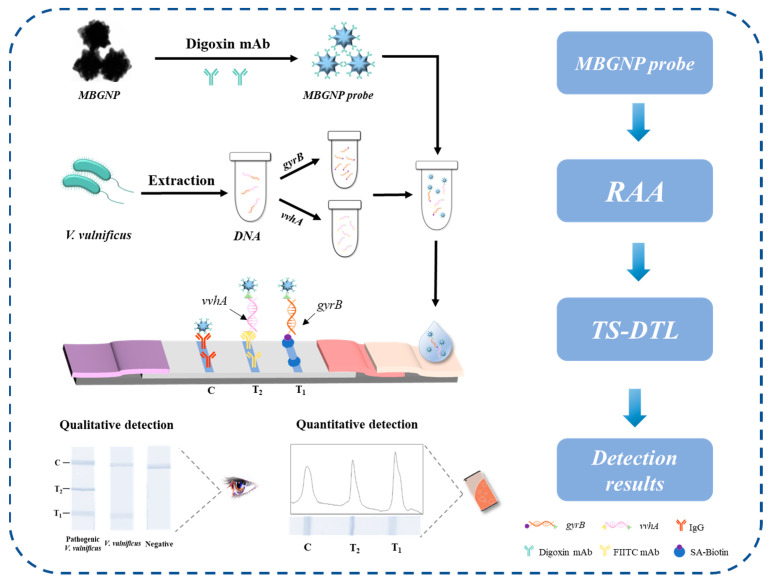
Schematic diagram of the rapid detection of *V. vulnificus* based on RAA-TS-DTL.

In this study, the virulent gene *vvhA* and the housekeeping gene *gyrB* of *V. vulnificus* were selected as the detection targets to develop an accurate and sensitive method of detecting the pathogenic *V. vulnificus*. As shown in Figure 1, digoxin and FAM were modified at the 5′ end of the *vvhA* primer, and digoxin and biotin were modified at the 5′ end of the *gyrB* primer. Their amplification products of RAA were used as samples for the TS-DTL assay. When the mixed solution of RAA products and the MBGNP probe was added to the sample pad of the TS-DTL, the biotin-modified *gyrB* could be captured by the SA on the T_1_ line during the flowing of the sample, the FAM-labeled *vvhA* was captured by the FITC mAb on T_2_ line, and digoxin mAb reacted with the GAM IgG secondary antibody on the C line. Thus, the MBGNP probes remained on the T_1_, T_2_ and C lines, resulting in a blue color on the T and C lines of the TS-DTL. By simple visual observation, rapid qualitative detection was achieved. Sensitive detection was realized by the intensity of the T_1_ and T_2_ lines collected with a smartphone and analyzed by Image J.

### 3.2. Characterization of the MBGNP and MBGNP Probe

**Figure 2:**
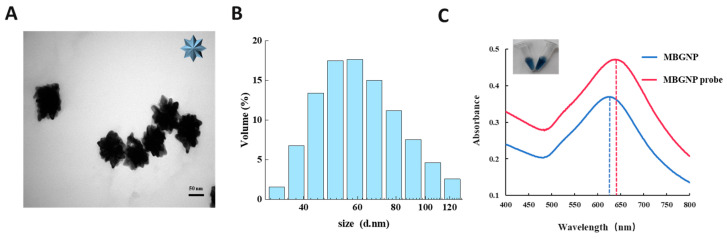
(**A**) TEM image of MBGNP. (**B**) DLS of MBGNP. (**C**) UV-vis absorption spectra of the MBGNP and MBGNP probe.

The morphology and particle size of the synthesized MBGNP were analyzed by TEM and DLS. TEM images showed that the MBGNP had an irregular shape with a large number of branches on the rough surface (Figure 2A). According to the results of DLS, the average hydrodynamic diameter of the MBGNP was about 69.67 nm with a low polydispersity coefficient (PDI) of 0.275 (Figure 2B), which indicated that the dispersibility of the MBGNP was good. In addition, the color of the MBGNP solution appeared blue, while the MBGNP probe solution was darker in color (Figure 2C). Moreover, the maximum absorption peak at 630 nm of MBGNP was shifted to 645 nm (MBGNP probe) when the MBGNP was coupled with digoxin mAb, indicating that the MBGNP probe was successfully prepared.

### 3.3. Screening of Optimal Primer Pairs for RAA

**Figure 3:**
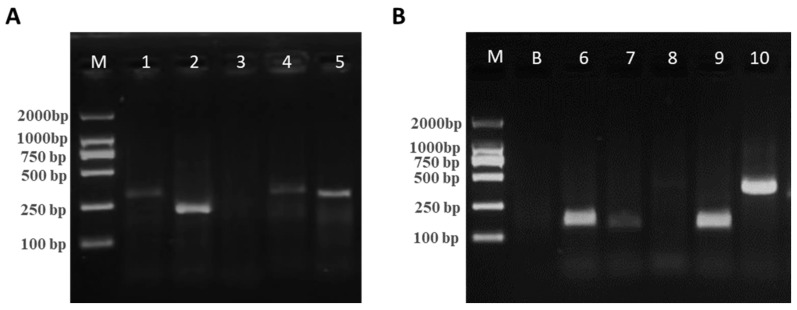
AGE results of RAA amplicons from the *vvhA* gene (**A**) and the *gyrB* gene. (**B**) Primer pairs. M, marker; B, blank sample; 1–10, primer Pairs 1–10.

To obtain the optimal primers, 10 pairs of primers were individually designed for *vvhA* and *gyrB* genes (Table 2 and Table 3) and analyzed by 2% AGE. As shown in Figure 3, the bands of the *vvhA* gene primer Pair 2 (P2) were observed to be darker and thicker, indicating a high concentration and the good amplification efficiency of RAA. In addition, the bands of *gyrB* gene primer Pair 10 (P10) were thick and easy to distinguish from the bands of the Group 2 primers due to the large molecular weight. Therefore, pairs from Group 2 and Group 10 were selected as the primers for RAA and the subsequent TS-DTL assay of *V. vulnificus*.

### 3.4. Optimization of the RAA-TS-DTL System

**Figure 4:**
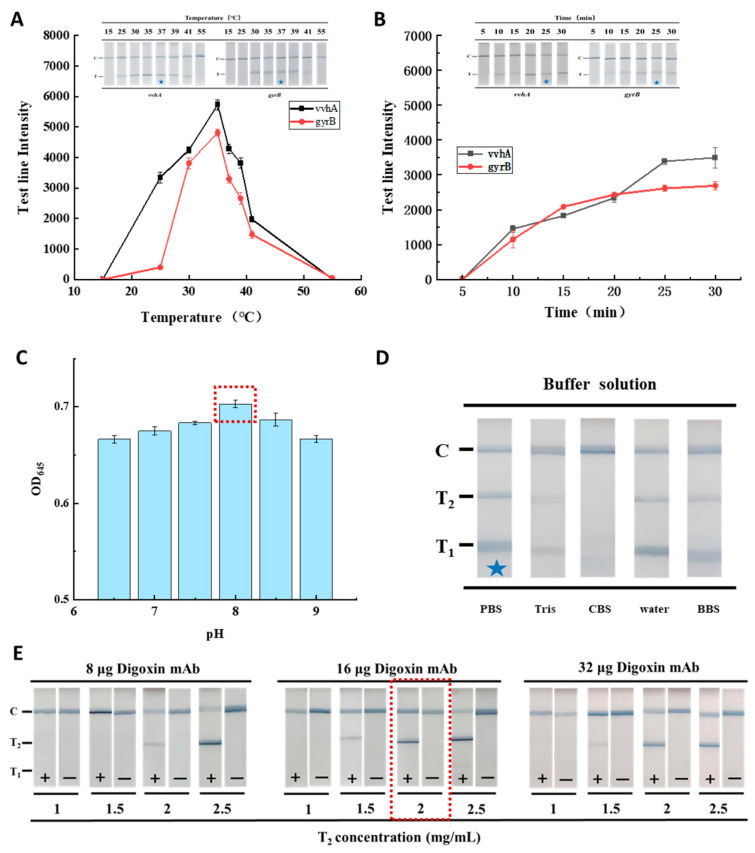
Optimization of RAA-TS-DTL. (**A**) Results of different RAA amplicons obtained at different temperatures (15–55 °C). (**B**) Results of different RAA amplicons obtained at different times (5–30 min). (**C**) Results of different pH values of the MBGNP probe. (**D**) Results of different buffer solutions of the TS-DTL. (**E**) Results of different concentrations of FITC mAb (1–2.5 mg/mL) on the T_2_ line and amounts of digoxin mAb (8–32 μg) coupled to the MBGNP. The red dashed boxes and the blue stars indicate the optimal conditions.

The results of optimizing RAA are shown in Figure 4A,B. The intensity of the T line of the *vvhA* and *gyrB* genes was found to be highest at 37 °C (Figure 4A), and was clearly visible (marked by blue asterisk) to the naked eye. Moreover, it was found that the intensity of the T_1_ and T_2_ lines increased with an increase in the detection time, and both genes tended to be saturated after 25 min (Figure 4B). Therefore, 37 °C and 25 min were selected as the optimal conditions for RAA. The optimization of the pH of the MBGNP probe is shown in Figure 4C. At pH 8.0, the absorbance value at 645 nm of the probe was found to be highest; therefore, pH 8.0 was optimal for preparation of the MBGNP probe.

The binding efficiency between the antibody and antigen on the test strip was found to be affected by the ionic strength of the chromatographic buffer solutions, the amount of coupled mAb on the probe and the concentration of the coating on the T lines [14]. Therefore, different types of buffer solution and digoxin mAb labeled on the MBGNP were optimized for the RAA-TS-DTL of *V. vulnificus*. As shown in Figure 4D, strong and clear T_1_, T_2_ and C lines were observed in 20 mM PBS (marked by a blue asterisk), whereas both T lines were invisible in 0.05 M CBS, and the intensity of the T_2_ line was less strong in other three kinds of buffer than in PBS. On the other hand, it was found that when the MBGNP probes were coupled with 16 or 32 μg of digoxin mAb, the color of the T lines was clearest in the positive sample tested by the TS-DTL coated with 2 mg/mL of FITC antibody at the T_2_ line (Figure 4E). According to the combination of detection performance and cost, PBS (20 mM, pH 7.4) and 16 μg digoxin mAb were selected as the optimal conditions for the RAA-TS-DTL of *V. vulnificus*.

### 3.5. Detection Performance of the RAA-TS-DTL System

#### 3.5.1. Specificity

**Figure 5:**
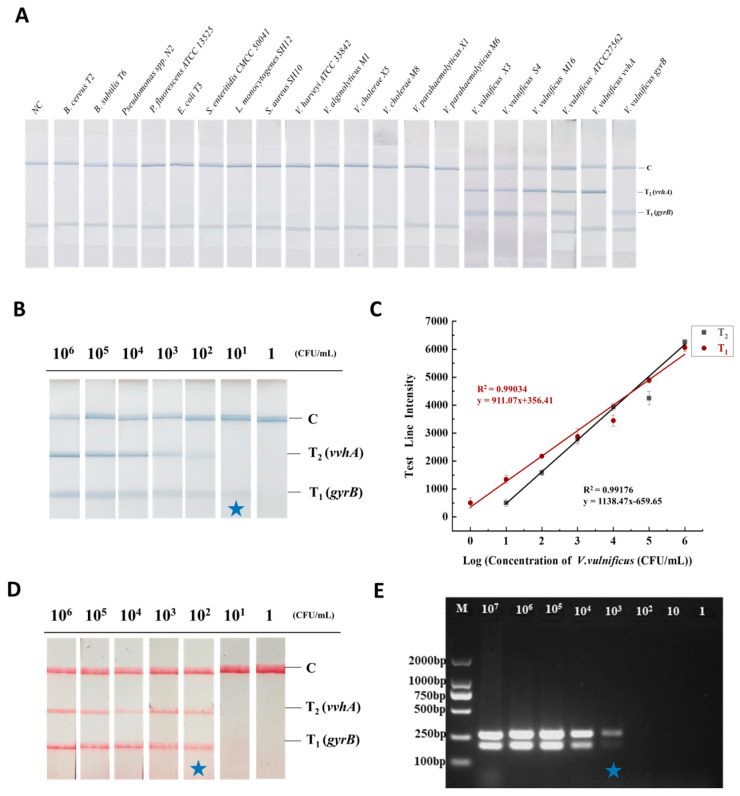
Evaluation of the performance of the RAA-TS-DTL and other methods for different concentrations of *V. vulnificus* (ATCC 27562). (**A**) Specificity results for 18 bacterial strains. (**B**) Qualitative detection results of RAA-TS-DTL. (**C**) Quantitative standard curves of T_1_ (*gyrB* gene) and T_2_ (*vvhA* gene). (**D**) Qualitative detection results of RAA combined with a commercial test strip. (**E**) AGE analysis of PCR products of the DNA from oyster samples spiked with different concentrations of *V. vulnificus*. NC, negative control prepared from ultrapure water instead of the DNA templates. The blue stars indicate the detection limits of different methods.

Under optimized detection conditions, 18 bacterial species (Table 1), including one standard strain of *V. vulnificus* (ATCC 27562), three strains of *V. vulnificus* isolated from aquatic products, six strains of *Vibrio* and eight strains of other species were used to evaluate the specificity of the established RAA-TS-DTL. As shown in Figure 5A, when the single *vvhA* or *gyrB* gene-amplified RAA products were used as the sample, only the corresponding T_1_ or T_2_ line was observed. However, both the T_1_ and T_2_ lines were observed in four strains of *V. vulnificus* (ATCC 27562, X3, S4 and M16). The *vvhA* gene is a specific virulence gene, and thus the developed RAA-TS-DTL method was specific for the pathogenic *V. vulnificus*. It was found that the predominant microorganisms in raw oysters were *Pseudomonas* (~22%) and *Vibrio* (~20%) [18]. However, both T lines were invisible in the other 14 bacterial strains, including 6 *Vibrio* spp., 2 *Pseudomonas* spp. and 6 other bacteria. These results suggested that the RAA-TS-DTL had good specificity for the detection of *V. vulnificus*.

#### 3.5.2. Sensitivity of RAA-TS-DTL for *V. vulnificus* in Oyster

None of the sterilized oyster homogenates grew colonies on LB agar, indicating that the oysters were successfully sterilized, and the amount of bacterial solution added was the content of *V. vulnificus* in the samples. Oyster samples spiked with serial dilutions of *V. vulnificus* ATCC 27562 were used to analyze the sensitivity of the RAA-TS-DTL detection system. As shown in Figure 5B, it was found that the color of the double T lines gradually became lighter with the decrease in concentration the of *V. vulnificus*. The T_1_ (*gyrB*) and T_2_ (*vvhA*) lines were invisible when the concentration of *V. vulnificus* was 1 CFU/mL and 10 CFU/mL, respectively. Therefore, the qualitative sensitivity was determined to be 10 CFU/mL for detection *of the gyrB* gene, and 100 CFU/mL for detecting the *vvhA* gene of *V. vulnificus*. Furthermore, it was found that the linear relationship between the concentration of *V. vulnificus* and the intensity of T_1_ was in the range of 1–10^6^ CFU/mL, while the linear relationship with the intensity of T_2_ was ranged from 10 to 10^6^ CFU/mL (Figure 5C). According to the formula G_m_ = G_0_ + 3 × SD and the fitting regression equation of the quantitative curve against the T_1_ and T_2_ lines, the LOD was calculated to be 6 CFU/mL for detecting the *gyrB* gene and 23 CFU/mL for detecting the *vvhA* gene of *V. vulnificus* in oyster. In addition, the same batch of RAA amplification products was also tested with a commercial test strip, and its qualitative sensitivity was found to be 10^2^ CFU/mL (Figure 5D). Moreover, the same batch of DNA samples of different concentrations of *V. vulnificus* was validated by PCR combined with AGE analysis. The bands of *vvhA* and *gyrB* were invisible for 10^2^ CFU/mL of *V. vulnificus* (Figure 5E), and thus the sensitivity of PCR-AGE was determined to be 10^3^ CFU/mL for the same batch of samples. Higher sensitivity was achieved for the developed RAA-TS-DTL, which was about five times higher than that of the commercial test strip and about 50 times higher than the PCR-AGE method.

#### 3.5.3. Accuracy

In this study, three types of aquatic products, including crucian carp (*Tilapia*), white prawn (*Litopenaeus vannamei*) and oyster (*Crassostrea gigas*), were individually spiked with the pathogenic *V. vulnificus* pretreated with PMAxx and used as samples for an assessment of the accuracy. The same batch of each real food sample was detected individually by the developed RAA-TS-DTL, a traditional culture method and PCR-AGE. As shown in Table 4, it was found that all fish samples spiked with *V. vulnificus* were detected by the traditional culture method (*n* = 18), while two samples were missed by PCR. This might be due to the low amount of bacterial DNA extracted from the sample and its insufficient sensitivity.

For the spiked shrimp samples (*n* = 25), the traditional culture and RAA-TS-DTL methods had the same detection rate of 96% (24/25), while the PCR method had a lower detection rate of 92% (23/25). It was found that one oyster sample was missed by all three methods, which might be due to the low concentration of spiked *V. vulnificus* in one sample. Overall, the detection rate of RAA-TS-DTL was 94% for all spiked samples (*n* = 50) and 100% for all negative samples (*n* = 10). The consistency between RAA-TS-DTL and the traditional culture method was 97.9%, indicating that RAA-TS-DTL had a high accuracy for *V. vulnificus* in foods. In general, the detection of *V. vulnificus* based on the traditional culture method takes a long time, about 3–5 days. However, the entire detection time of the developed RAA-TS-DTL method, including time for preparing the sample, was only about 50 min.

## 4. Discussion

The accumulation of *V. vulnificus* in mollusks such as oyster poses a major threat to the aquaculture industry and public health. The onset of disease in humans occurs within 12 h to 3 days after infection with *V. vulnificus*, with a rapid onset and high mortality rates [19]. Therefore, a rapid, sensitive and accurate detection method would be beneficial for reducing the risk of *V. vulnificus*. On the basis of isothermal amplification technology, researchers have developed rapid and sensitive LAMP [20], RAA combined with CRISPR [13], RPA combined with test strips [11,21] and other methods of detecting *V. vulnificus*. However, these methods all target a single gene to achieve the specific detection of *V. vulnificus*. In general, when a conserved gene is selected as a target, it is difficult to distinguish whether it is from the pathogenic *V. vulnificus* or not. If both a conserved gene and a virulence gene are used as targets, it can be more accurate for identifying or determining pathogenic bacteria than a single virulence gene target.

In this study, a rapid RAA-TS-DTL method for *V. vulnificus* was developed based on dual genes by combining isothermal amplification technology with a test strip, in which the housekeeping gene *gyrB* was detected by the T_1_ line and the virulence *vvhA* gene was detected by the T_2_ line of the test strip. It was found that the established RAA-TS-DTL method could observe the double T_1_ and T_2_ lines for all four strains of *V. vulnificus* (one standard strain and three aquatic product isolates), while the T lines were invisible during the detection of other *Vibrio* spp. (*n* = 6), *Pseudomonas* spp. (*n* = 2) and other pathogens (*n* = 6) separated from aquaculture water and aquatic products (Figure 5A). All four strains of *V. vulnificus* were verified to carry the *vvhA* gene by PCR-AGE with the reported primers for the *vvhA* gene, achieving the target bands. These results indicated that the developed RAA-TS-DTL was specific for pathogenic *V. vulnificus*. The target region of the *gyrB* gene in this study contains a number of different alleles. Therefore, with our RAA-TS-DTL system, it was difficult to ensure the detection of all *V. vulnificus* bacteria in the environment or foods. It is possible that the T_2_ and C lines on the test strip could be visible but the T_1_ lines might not be visible. To this condition, a retest for *V. vulnificus* using other methods such as the traditional culture method is recommended.

In addition, the sensitive detection of *V. vulnificus* in oysters was realized by RAA-TS-DTL. As shown in Table 5, the classical qPCR, multiple PCR and droplet digital PCR (ddPCR) methods for *V. vulnificus* had LODs ranging from 10 to 100 CFU/mL [8,22,23]. The RAA-TS-DTL developed on the basis of the *gyrB* gene could detect *V. vulnificus* in oyster at levels as low as 6 CFU/mL, which was 15 times more sensitive than that of the qPCR method established by D’Souza et al. (100 CFU/mL) [8]. Moreover, it could detect *V. vulnificus* in oyster at 23 CFU/mL on the basis of the *vvhA* gene, which was slightly more sensitive than the RPA-LFD method using a commercial test strip established by Park et al. (30 CFU/mL) [21]. This might be due to the larger specific surface area of MBGNP [15], resulting in the coupling of more antibodies on the probes. Although the LOD of our method was close to that of LAMP [10,24,25] or RAA–CRISPR [26,27], it did not require fluorescence excitation equipment, as is the case for the CRISPR method, and it could achieve isothermal amplification at lower temperatures (such as 37 °C) than the LAMP method. Moreover, the sensitivity of RAA-TS-DTL was similar with that of ICTS through the principle of binding of the antigen and antibody [7].

The detection performance of the test strip method, such as sensitivity, is susceptible to interference from the food matrix [28]. The results of this study for *V. vulnificus* in three food products, namely fish, shrimp and oyster, showed that the RAA-TS-DTL method had a detection rate of 94% for 50 spiked food samples examined blindly. The consistency between RAA-TS-DTL and the traditional culture method was 97.9% (*n* = 50, Table 4), showing good accuracy for *V. vulnificus* in foods. Moreover, the entire RAA-TS-DTL system, including preparation of the RAA sample and detection with the test strip, could be completed within 50 min, which was significantly faster than the ELISA (~6 h) [6] and the PCR methods (~1.5 h) [29] for *V. vulnificus*. Overall, the developed RAA-TS-DTL method was rapid, simple and sensitive for detecting pathogenic *V. vulnificus* in aquatic products. It provides a new ideal for rapid screening or on-site detection of *V. vulnificus* in aquatic products and aquaculture water.

## Figures and Tables

**Table 1:** Bacterial strains used in this study.

Number	Bacterial Strains	Species and Strain	Source
1	*Vibrio vulnificus*	*Vibrio vulnificus* ATCC27562	GDMCC
2		*Vibrio vulnificus* X3	Isolated from shrimp
3		*Vibrio vulnificus* S4	Isolated from scallop
4		*Vibrio vulnificus* M16	Isolated from oysters
5	Other *Vibrio* spp.	*Vibrio parahaemolyticus* M6	Isolated from oysters
6		*Vibrio parahaemolyticus* X1	Isolated from shrimp
7		*Vibrio cholerae* M8	Isolated from oysters
8		*Vibrio cholerae* X5	Isolated from shrimp
9		*Vibrio alginolyticus* M1	Isolated from oysters
10		*Vibrio harveyi* ATCC 33842	GDMCC
11	Other bacterial strains	*Staphylococcus aureus* SH10	Preserved in our laboratory
12		*Listeria monocytogenes* SH12	
13		*Salmonella enteritidis* CMCC 50041	CMCC
14		*Escherichia coli* T3	Isolated from aquaculture water
15		*Pseudomonas fluorescens* ATCC 13525	GDMCC
16		*Pseudomonas* spp. N2	Isolated from milk
17		*Bacillus subtilis* T6	Isolated from aquaculture water
18		*Bacillus cereus* T2	Isolated from aquaculture water

**Table 2:** Primers of the *vvhA* gene of *V. vulnificus*.

Assay	Number	Name	Sequence (5′–3′) and Modification	Amplicon Size (bp)
Basic RAA(*vvhA*)	1	F1	GATACTTACGGTTACTCCATCGGTATTAAC	300
		R1	GATTGGGTTGAACTTCGTCTTATCAAATAC	
	2	F2	GCGGAAGTGAACAAAGACGGCCCGAAAGT	215
		R2	CAGTGAGCGGCGGTGAAATAGCATCCAAGC	
	3	F3	GAAGTCAGTGGTCATTTACAACTACTC	205
		R3	CGTCATAGTTCGGTTTGAAGTTGGAATAAGAG	
	4	F4	ACTTACATTGGCCCATTCGCCAGCAGTTAT	278
		R4	GATGAGCGGTTGTTGATGCGATAGTCTTTT	
	5	F5	CTCATTTACTTACAACTACTCGAAAACCTTG	238
		R5	ATAGTTCGGTTTGAAGTTGGAATAAGAGATTG	
RAA-TS-DTL(*vvhA*)		F2-FAM	FAM-GCGGAAGTGAACAAAGACGGCCCGAAAGTG	215
		R2-Dig	Digoxin-CAGTGAGCGGCGGTGAAATAGCATCCAAGC	
PCR		P-F1	TTCCAACTTCAAACCGAACTATGA	205
		P-R1	ATTCCAGTCGATGCGAATACGTTG	

**Table 3:** Primers of the *gyrB* gene of *V. vulnificus*.

Assay	Number	Name	Sequence (5′–3′) and Modification	Amplicon Size (bp)
Basic RAA (*gyrB*)	6	F6	CCGTAAGAACCAAGCAATCCTACCGCTAAA	140
		R6	TGTACTCGTCACGACCGATACCACAACCTA	
	7	F7	ACAGCTACATGGACAAAGAAGGCTACTCGA	157
		R7	TTCACTTCACTAGAAACCAGTTTGTCTTTA	
	8	F8	GAAACCTTCACCAACATCGAATTTCATTAT	426
		R8	CAGTGAGCGGCGGTGAAATAGCATCCAAGC	
	9	F9	GAAACCTTCACCAACATCGAATTTCATTAT	138
		R9	TTCATACATGAAGTGATCTTTCTTATCTTCTT	
	10	F10	GCCAAACCAAAGACAAACTGGTTTCTAGTG	360
		R10	CTACGTTTAGAATCTTACCTTTTAGCGGTAGG	
RAA-TS-DTL (*gyrB*)		F10- Bio	Biotin-GCCAAACTAAAGACAAACTGGTTTCTAGTG	360
		R10-Dig	Digoxin-CTACGTTTAGAATCTTACCTTTTAGCGGTAGG	
PCR		P-F2	GTCCGCAGTGGAATCCTTCA	285
		P-R2	TGGTTCTTACGGTTACGGCC	

**Table 4:** Accuracy results of RAA-TS-DTL based on blind tests.

Sample Type	Number of Spiked Samples	Number of Negative Samples	Number of Samples Detected (+/−)		
			Traditional Culture	PCR-AGE	RAA-TS-DTL
Fish	18	2	18/2	16/2	17/2
Shrimp	25	5	24/5	23/5	24/5
Oyster	7	3	6/3	6/3	6/3

**Table 5:** Comparison of different detection methods for *V. vulnificus*.

Method	Target Gene	Sensitivity	Sample	References
ICTS	/	10 CFU/mL	oyster	[7]
qPCR	*gyrB*	100 CFU/mL	clam meat	[8]
Multiple PCR	*vvhA*	10 CFU /mL	cultured shrimps	[23]
ddPCR	*vvhA*	15.4 CFU/mL	plasma	[22]
LAMP	*gyrB*	10 fg/μL	culture solution	[10]
RPA-LFD	*vvhA*	30 CFU/mL	oyster	[21]
RAA-CRISPR	*vvhA*	20 CFU/mL	Seafood	[26]
RAA-TS-DTL	*vvhA* *gyrB*	23 CFU/mL 6 CFU/mL	oyster	This study

## Data Availability

The data used to support the findings of this study can be made available by the corresponding author upon request.

## Supplementary Materials

The following supporting information can be downloaded at https://www.mdpi.com/article/10.3390/foods12193605/s1, Figure S1. Comparative sequences of *gyrB* from *V. vulnificus*. Figure S2. Partial BLAST results of the primers. (A) Primer Pair 10 of the *gyrB* gene. (B) Primer Pair 2 of the *vvhA* gene. Figure S3. BLAST results of the *gyrB* (A) and *vvhA* (B) target sequences from the NCBI database.
