# Supplementary material for: A Novel RAA Combined Test Strip Method Based on Dual Gene Targets for Pathogenic Vibrio vulnificus in Aquatic Products
Source: Foods. 2023 Sep 28;12(19):3605. doi: 10.3390/foods12193605 (PMC10572794; doi:10.3390/foods12193605)
Supplement: Supplementary file 1 [file foods-12-03605-s001.zip › foods-2572210-supplementary.pdf]

Supplementary Materials

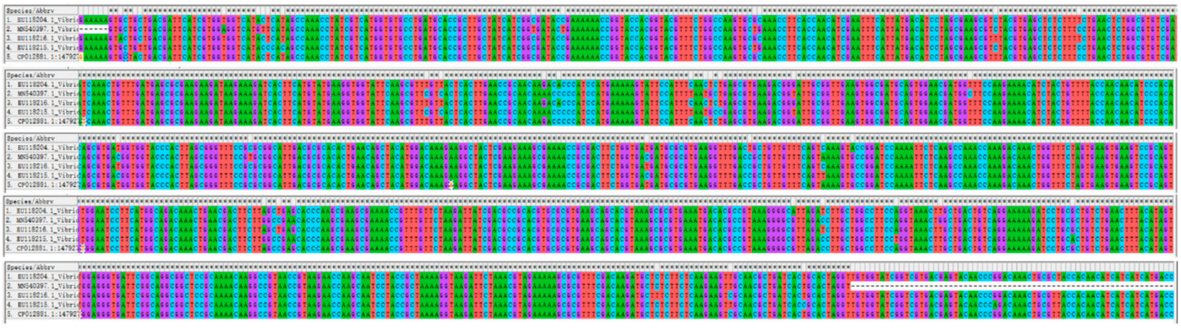

Figure S1. Comparative sequences of *gyrB* from *V. vulnificus*.

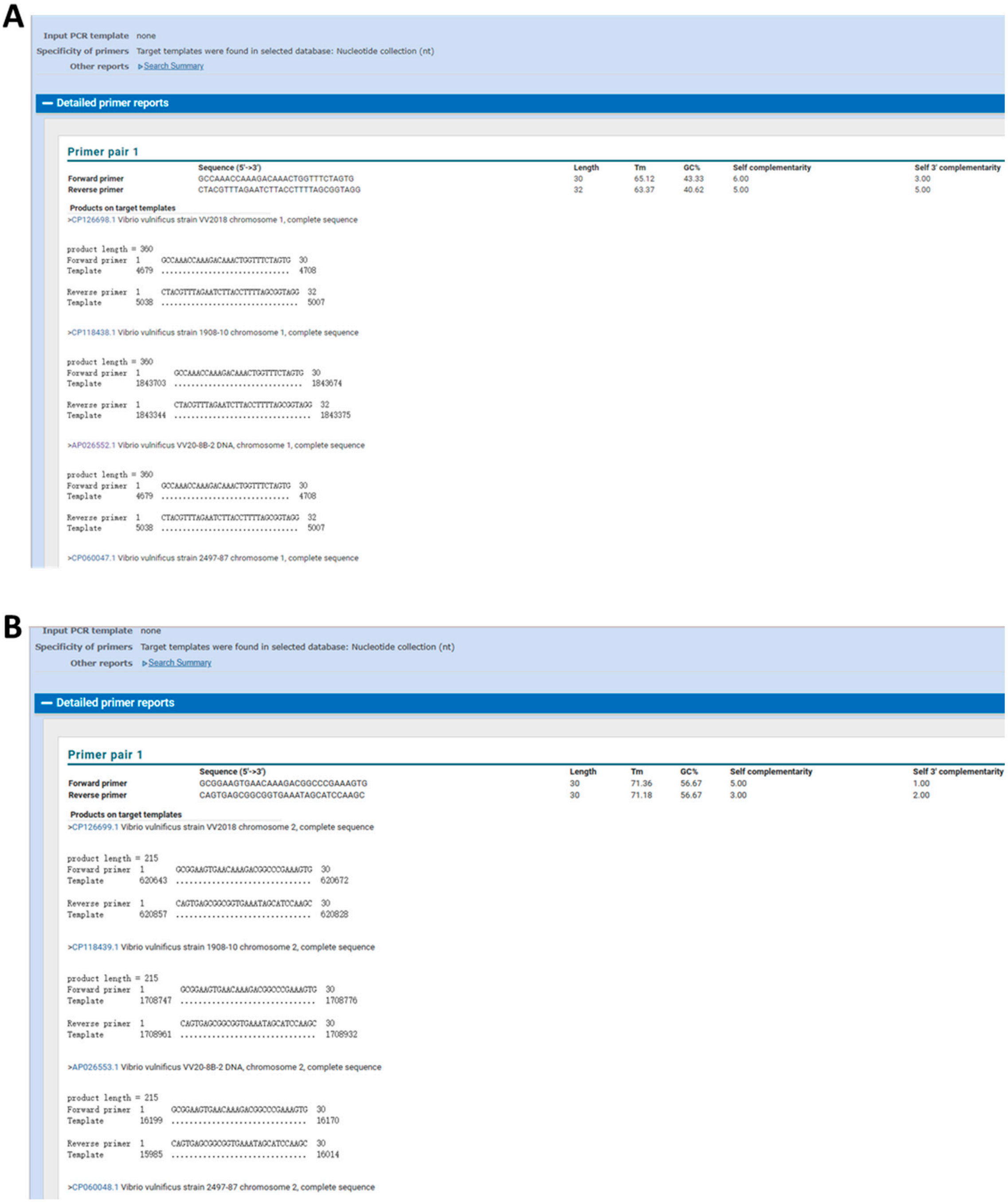

Figure S2. Primer-BLAST partial results. (A) primer pair 10 of *gyrB* gene. (B) primer pair 2 of *vvhA* gene

A

select all 100 sequences selected — GenBank | Graphics | Distance tree of results | MSA Viewer

Description	Scientific Name	Max Score	Total Score	Query Cover	E value	Per. Ident	Acc. Len	Accession
Vibrio vulnificus strain FDAARGOS_663 chromosome 2, complete sequence	Vibrio vulnificus	665	665	100%	0.0	100.00%	3249353	CP044069.1
Vibrio vulnificus NBRC 15645 = ATCC 27562 chromosome 1, complete sequence	Vibrio vulnificus NBRC 15645 = ATCC ...	665	665	100%	0.0	100.00%	3266118	CP012881.1
Vibrio vulnificus strain 2142-77 chromosome 1, complete sequence	Vibrio vulnificus	660	660	100%	0.0	99.72%	3294783	CP035731.1
Vibrio vulnificus Env1 chromosome I, complete sequence	Vibrio vulnificus Env1	660	660	100%	0.0	99.72%	3241343	CP017635.1
Vibrio vulnificus strain ENV-1 DNA gyrase beta subunit (gyrB) gene, partial cds	Vibrio vulnificus Env1	660	660	100%	0.0	99.72%	1195	EU118207.1
Vibrio vulnificus strain 395R DNA gyrase beta subunit (gyrB) gene, partial cds	Vibrio vulnificus	660	660	100%	0.0	99.72%	1196	EU118199.1
Vibrio vulnificus strain 06-2410 chromosome 1, complete sequence	Vibrio vulnificus	654	654	100%	0.0	99.44%	3225605	CP046832.1
Vibrio vulnificus strain FORC_077 chromosome 1, complete sequence	Vibrio vulnificus	654	654	100%	0.0	99.44%	3217678	CP027030.1
Vibrio vulnificus strain FDAARGOS_119 chromosome 2, complete sequence	Vibrio vulnificus	654	654	100%	0.0	99.44%	3236438	CP014049.2
Vibrio vulnificus strain FORC_054 chromosome 1, complete sequence	Vibrio vulnificus	654	654	100%	0.0	99.44%	3311092	CP019121.1
Vibrio vulnificus strain CECT 4999 chromosome I, complete sequence	Vibrio vulnificus	654	654	100%	0.0	99.44%	3394464	CP014636.1
Vibrio vulnificus strain 93U204 chromosome I, complete sequence	Vibrio vulnificus	654	654	100%	0.0	99.44%	3315989	CP009261.1
Vibrio vulnificus strain SS108C 5C1 DNA gyrase beta subunit (gyrB) gene, partial cds	Vibrio vulnificus	654	654	100%	0.0	99.44%	1198	EU118216.1
Vibrio vulnificus strain 301 1A1 DNA gyrase beta subunit (gyrB) gene, partial cds	Vibrio vulnificus	654	654	100%	0.0	99.44%	1187	EU118201.1
Vibrio vulnificus strain 300 1C1 DNA gyrase beta subunit (gyrB) gene, partial cds	Vibrio vulnificus	654	654	100%	0.0	99.44%	1203	EU118200.1
Vibrio vulnificus strain ATCC 27562 gyrase B subunit (gyrB) gene, partial cds	Vibrio vulnificus NBRC 15645 = ATCC ...	654	654	100%	0.0	99.44%	1174	AY705491.1
Vibrio vulnificus strain VVSF186 DNA gyrase beta subunit (gyrB) gene, partial cds	Vibrio vulnificus	649	649	100%	0.0	99.17%	1041	MN540398.1
Vibrio vulnificus strain VVSF184 DNA gyrase beta subunit (gyrB) gene, partial cds	Vibrio vulnificus	649	649	100%	0.0	99.17%	1041	MN540397.1
Vibrio vulnificus strain 2009V-1035 chromosome 1	Vibrio vulnificus	649	649	100%	0.0	99.17%	3317415	CP035784.1
Vibrio vulnificus strain FORC_037 chromosome I, complete sequence	Vibrio vulnificus	649	649	100%	0.0	99.17%	3224939	CP016321.1
Vibrio vulnificus strain 2497-87 chromosome 1, complete sequence	Vibrio vulnificus	649	649	100%	0.0	99.17%	3260230	CP060047.1
Vibrio vulnificus strain ATCC 43382 DNA gyrase beta subunit (gyrB) gene, partial cds	Vibrio vulnificus	649	649	100%	0.0	99.17%	1214	EU118204.1

B

select all 95 sequences selected — GenBank | Graphics | Distance tree of results | MSA Viewer

Description	Scientific Name	Max Score	Total Score	Query Cover	E value	Per. Ident	Acc. Len	Accession
Vibrio vulnificus strain 06-2410 chromosome 2, complete sequence	Vibrio vulnificus	398	398	100%	3e-106	100.00%	1771136	CP046833.1
Vibrio vulnificus strain 2009V-1035 chromosome 2, complete sequence	Vibrio vulnificus	398	398	100%	3e-106	100.00%	1698490	CP035783.1
Vibrio vulnificus strain VvMBC99 cytolysin (vvhA) gene, partial cds	Vibrio vulnificus	398	398	100%	3e-106	100.00%	763	KF255387.1
Vibrio vulnificus strain VvMBC58 cytolysin (vvhA) gene, partial cds	Vibrio vulnificus	398	398	100%	3e-106	100.00%	763	KF255356.1
Vibrio vulnificus strain VvMBC55 cytolysin (vvhA) gene, partial cds	Vibrio vulnificus	398	398	100%	3e-106	100.00%	763	KF255353.1
Vibrio vulnificus strain VvMBC26 cytolysin (vvhA) gene, partial cds	Vibrio vulnificus	398	398	100%	3e-106	100.00%	763	KF255334.1
Vibrio vulnificus strain VvMBC31 cytolysin (vvhA) gene, partial cds	Vibrio vulnificus	398	398	100%	3e-106	100.00%	763	KF255339.1
Vibrio vulnificus strain AM46407 cytolysin (vvhA) gene, partial cds	Vibrio vulnificus	398	398	100%	3e-106	100.00%	763	KF255332.1
Vibrio vulnificus strain AM41394 cytolysin (vvhA) gene, partial cds	Vibrio vulnificus	398	398	100%	3e-106	100.00%	763	KF255322.1
Vibrio vulnificus strain 2497-87 chromosome 2, complete sequence	Vibrio vulnificus	398	398	100%	3e-106	100.00%	1772589	CP060048.1
Vibrio vulnificus isolate IF Vv10 cytolysin (vvhA) gene, partial cds	Vibrio vulnificus	180	180	45%	1e-40	100.00%	659	AF376032.1
Vibrio vulnificus isolate IF Vv24 cytolysin (vvhA) gene, partial cds	Vibrio vulnificus	180	180	45%	1e-40	100.00%	656	AF376031.1
Vibrio vulnificus isolate CNRVC 970121 cytolysin (vvhA) gene, partial cds	Vibrio vulnificus	167	167	41%	1e-36	100.00%	623	AF376029.1
Vibrio vulnificus strain CIP 75.4T cytolysin (vvhA) gene, partial cds	Vibrio vulnificus ...	167	167	41%	1e-36	100.00%	622	AF376027.1
Vibrio vulnificus isolate IF Vv18 cytolysin (vvhA) gene, partial cds	Vibrio vulnificus	165	165	41%	4e-36	100.00%	651	AF376030.1
Vibrio vulnificus isolate IF Vv11 cytolysin (vvhA) gene, partial cds	Vibrio vulnificus	163	163	40%	1e-35	100.00%	640	AF376033.1
Vibrio vulnificus isolate CNRVC 970120 cytolysin (vvhA) gene, partial cds	Vibrio vulnificus	132	132	33%	4e-26	100.00%	603	AF376028.1
Vibrio vulnificus strain 628-7 cytotoxin (vvhA) gene, partial cds	Vibrio vulnificus	115	115	28%	4e-21	100.00%	576	JF682556.1
Vibrio vulnificus strain CMFRI/VV-08 VvhA gene, partial cds	Vibrio vulnificus	73.1	73.1	18%	2e-08	100.00%	462	MW132718.1
Vibrio vulnificus strain CMFRI/VV-06 VvhA gene, partial cds	Vibrio vulnificus	73.1	73.1	18%	2e-08	100.00%	468	MW132717.1
Vibrio vulnificus strain CMFRI/VV-05 VvhA gene, partial cds	Vibrio vulnificus	71.3	71.3	17%	8e-08	100.00%	461	MW132716.1
Vibrio vulnificus haemolysin A (vvhA) gene, partial cds	Vibrio vulnificus	69.4	69.4	17%	3e-07	100.00%	463	MH357338.1
Vibrio vulnificus strain CMFRI/VV-10 VvhA gene, partial cds	Vibrio vulnificus	69.4	69.4	17%	3e-07	100.00%	463	MW195530.1

Figure S3. BLAST results of *gyrB* (A) and *vvhA* (B) target sequences from NCBI database.
